# 7T magnetic resonance imaging-based investigation of the correlation between mammillary body structure and cognitive impairment in patients with spinocerebellar ataxia type 3

**DOI:** 10.1093/psyrad/kkaf010

**Published:** 2025-06-19

**Authors:** Congwei Li, Yunsong Peng, Peiling Ou, Ru Wen, Wei Chen, Chong Tian, Zhiming Zhen, Xingang Wang, Lan Ou, Chen Liu, Bijia Wang

**Affiliations:** 7T Magnetic Resonance Imaging Translational Medical Center, Department of Radiology, Southwest Hospital, Army Medical University (Third Military Medical University), Chongqing 400038, China; Guizhou Province International Science and Technology Cooperation Base for Precision Imaging Diagnosis and Treatment, Key Laboratory of Advanced Medical Imaging and Intelligent Computing of Guizhou Province, Department of Radiology, Guizhou Provincial People's Hospital, Guiyang, Guizhou 550002, China; 7T Magnetic Resonance Imaging Translational Medical Center, Department of Radiology, Southwest Hospital, Army Medical University (Third Military Medical University), Chongqing 400038, China; 7T Magnetic Resonance Imaging Translational Medical Center, Department of Radiology, Southwest Hospital, Army Medical University (Third Military Medical University), Chongqing 400038, China; MR Research Collaboration Team, Siemens Healthineers Ltd, Guangzhou 510000, China; Department of Radiology, Guizhou Provincial People's Hospital, Guiyang 550002, China; 7T Magnetic Resonance Imaging Translational Medical Center, Department of Radiology, Southwest Hospital, Army Medical University (Third Military Medical University), Chongqing 400038, China; 7T Magnetic Resonance Imaging Translational Medical Center, Department of Radiology, Southwest Hospital, Army Medical University (Third Military Medical University), Chongqing 400038, China; 7T Magnetic Resonance Imaging Translational Medical Center, Department of Radiology, Southwest Hospital, Army Medical University (Third Military Medical University), Chongqing 400038, China; 7T Magnetic Resonance Imaging Translational Medical Center, Department of Radiology, Southwest Hospital, Army Medical University (Third Military Medical University), Chongqing 400038, China; Department of Neurology, Southwest Hospital, Army Medical University (Third Military Medical University), Chongqing 400038, China

**Keywords:** spinocerebellar ataxia, cognitive impairment, Papez circuit, 7T MRI, mammillary body

## Abstract

**Background:**

Spinocerebellar ataxia type 3 (SCA3) is a hereditary disease characterized by cerebellar atrophy and motor dysfunction. Patients also exhibit non-ataxic symptoms such as cognitive impairment. While prior neuroimaging studies have identified multiple cognition-associated brain regions in SCA3 patients, research on Papez circuit structural damage (e.g., mammillary bodies (MBs)) remains sparse. Advancements in 7T magnetic resonance imaging (MRI) technology have enabled scanning and quantitative analysis of structures such as the MBs within the Papez circuit. In this study, we investigated the relationship between cognitive impairment in patients with SCA3 and structural changes in the three Papez circuit structures: the MBs, the mammillothalamic tract (MTT), and the post-commissural fornix (PF).

**Methods:**

This cross-sectional study included 46 SCA3 patients and 48 healthy controls undergoing 7T MRI and neuropsychological assessments. Using manual delineation and a deep learning model, we extracted the MB, MTT, and PF volumes from participants. Subsequently, we statistically analyzed the quantitative data.

**Results:**

SCA3 patients exhibited reduced MB, PF, and MTT volumes compared with those of the healthy controls. The MB, left MTT, and left PF volumes were significantly lower in cognitive impairment than in cognitive preserved. Cognitive function in SCA3 patients was positively correlated with the MB, left MTT, and left PF, whereas motor function was negatively correlated with the MB and left PF.

**Conclusion:**

Decreased cognitive and memory function in SCA3 patients is associated with MB, MTT, and PF alterations and is more pronounced on the left side. Motor dysfunction may be correlated with cognitive impairment development.

## Introduction

Spinocerebellar ataxias (SCAs) are a group of neurogenetic disorders characterized by degeneration and atrophy of the spinal cord, cerebellum, and brainstem due to genetic mutations (Herrmann *et al*., 2019; Klockgether *et al*., 2019). SCAs typically manifest between the ages of 30 and 40 years, exhibit high genetic heterogeneity, are predominantly inherited in an autosomal dominant pattern, and have high disability and mortality rates (Krygier & Mazurkiewicz-Bełdzińska, 2021). The primary clinical presentation of SCA type 3 (SCA3) is progressive cerebellar ataxia (Klockgether *et al*., 2019), and the disease is frequently associated with non-motor symptoms such as cognitive impairment (CI) (Kawai *et al.*, 2004; Garrard *et al., 2008*; Hengel *et al.*, 2023) and vision loss(Schöls *et al*., 2004; Braga-Neto *et al*., 2012; Chen *et al*., 2022). As SCA3 progresses, non-motor symptoms intensify, cognitive abilities decline progressively, and patients eventually lose the ability to live independently, underscoring the necessity for research into CI in these patients (Ishikawa *et al*., 2002).

SCA3 patients exhibit degenerative changes in brain structure and function. Structural imaging and autopsies of these patients have revealed that the primary sites of atrophy are the cerebellum, brainstem, substantia nigra, and globus pallidus (Buijsen *et al*., 2019). Consequently, cerebellar atrophy is considered the “core defect” of SCA3 (Rezende *et al*., 2018). Studies have also identified potential links between the cerebellum and cognitive and emotional processes (Buckner, 2013; Schmahmann *et al*., 2019; Sereno *et al*., 2020). The cerebellum may participate in cognitive functions through the frontocerebellar circuit that includes the cerebellum–parietal lobe–thalamocortical pathway (Liu *et al*., 2024; O'Callaghan *et al*., 2016; Witter & De Zeeuw, 2015). Previous studies on patients with SCA3 have shown that those with CI exhibit volume loss in the temporal, frontal, parietal, and insular brain regions (Silva *et al*., 2015) and in bilateral lobule VI, right lobule Crus I, and right lobule IV (Ye *et al*., 2024). However, in addition to the frontocerebellar circuit structure, the Papez circuit is an important core area affecting cognitive function (Papez, 1937). To date, no published studies have examined the Papez circuit and cognitive function in patients with SCA3. The Papez circuit refers to the neural pathway originating from the hippocampus; passing through the mammillary bodies (MBs), anterior thalamic nuclei, and cingulate gyrus; and returning to the hippocampus to form a closed loop (Papez *et al*., 1937; Bubb *et al*., 2017). The Papez circuit has been shown to be closely related to cognitive and memory functions in diseases such as Alzheimer's disease (AD) (Forno *et al*., 2021; Hari *et al*., 2023), multiple sclerosis (Marchesi *et al*., 2022), and alcohol use disorder (Morand *et al*., 2024).

The MBs are important nuclei in the ventral hypothalamus, connecting to the thalamus via the mammillothalamic tract (MTT) and to the hippocampus via the fornix. Therefore, the conduction structure formed by the MBs, MTT, and fornix is a crucial core structure within the Papez circuit (Aggleton & Sahgal, 1993; Bubb *et al*., 2017; Vann & Aggleton, 2004). On a cognitive and functional level, the MBs send information to the anterior temporal lobe network, and their structure and function are closely related to hippocampal integrity. They play crucial roles in regulating emotions, stress, anxiety, and memory functions. The MBs also play independent roles in memory distinct from those of the hippocampus (Vann & Aggleton, 2004). Therefore, studying the MBs, MTT, and fornix is necessary in investigating the CI mechanisms in patients with SCA3. However, owing to the small size and irregular shape of the MBs, MTT, and post-commissural fornix (PF) and the interference of cerebrospinal fluid (CSF), these structures are difficult to visualize on traditional magnetic resonance imaging (MRI). Therefore, research on the relationship between CI in SCA3 patients and structural changes in the Papez circuit remains limited. The increasing clinical application of 7T MRI offers significant advantages in distinguishing subtle subcortical nuclei that are invisible on traditional MRI. Diffusion tensor imaging (DTI) can map white matter tracts (e.g. MTT, fornix), but its limited spatial resolution (typically ≥2 mm isotropic) and susceptibility to partial volume effects hinder precise delineation of small nuclei. The 7T MP2RAGE (7 Tesla Magnetization Prepared 2 Rapid Acquisition Gradient Echoes) sequence can display structures that are invisible on traditional MRI (Choi *et al*., 2019). Its high spatial resolution and optimized contrast-to-noise ratio, which are essential for visualizing small Papez circuit structures, are often obscured by CSF partial volume effects in conventional sequences (Marques *et al*., 2010). This advancement enables quantitative structural study of the MBs, PF, and MTT.

Therefore, to investigate the relationship between the structural integrity of the MB, MTT, and PF in SCA3 patients and CI, we hypothesized that the MB, MTT, and PF structures within the Papez circuit are involved in CI in patients with SCA3 and are related to motor dysfunction. To test this hypothesis, we aimed to (i) demonstrate that the structural integrity of the MB, MTT, and PF in SCA3 patients is reduced compared with that of healthy individuals, (ii) show that SCA3 patients with CI have greater volume loss in the MB, MTT, and PF compared with those without CI, and (iii) demonstrate whether a relationship exists between dyskinesia and CI in SCA3 patients.

## Methods

### Clinical assessment

This was a prospective cross-sectional study exploring the correlation between CI in SCA3 patients and structural integrity of the MBs, MTT, and PF. Initially, 106 patients with SCA were recruited. All SCA3 participants were diagnosed through genetic analysis of peripheral blood samples, which identified cytosine–adenine–guanine (CAG) repeat expansions in their *ATXN3* alleles, with the number of repeats ranging from 56 to 87. We implemented rigorous quality control measures through triplicate verification, sequencing-based confirmation, and calibration with certified reference materials. The inclusion criteria were that patients (i) had a confirmed diagnosis of SCA3 through genetic testing; (ii) were aged between 18 and 65 years and were right-handed; (iii) underwent neuropsychological assessments using the Montreal Cognitive Assessment (MoCA; Beijing Version), digit span test (DST), rapid verbal retrieve (RVR), cerebellar cognitive affective syndrome (CCAS) scale, Scale for the Assessment and Rating of Ataxia (SARA), International Cooperative Ataxia Rating Scale (ICARS), Activity of Daily Living Scale (ADL), Instrumental Activities of Daily Living Scale (IADL), and Hamilton Depression Rating Scale (HAMD); and (iv) agreed to and could tolerate 7T MRI scanning. The exclusion criteria were (i) contraindications to MRI, such as claustrophobia, cardiac stents, cochlear implants, or other implants that may affect MRI; (ii) history of cranial or neurological disorders; and (iii) poor-quality MRI images. After screening, 46 patients met the criteria and were included.

**Figure 1:**
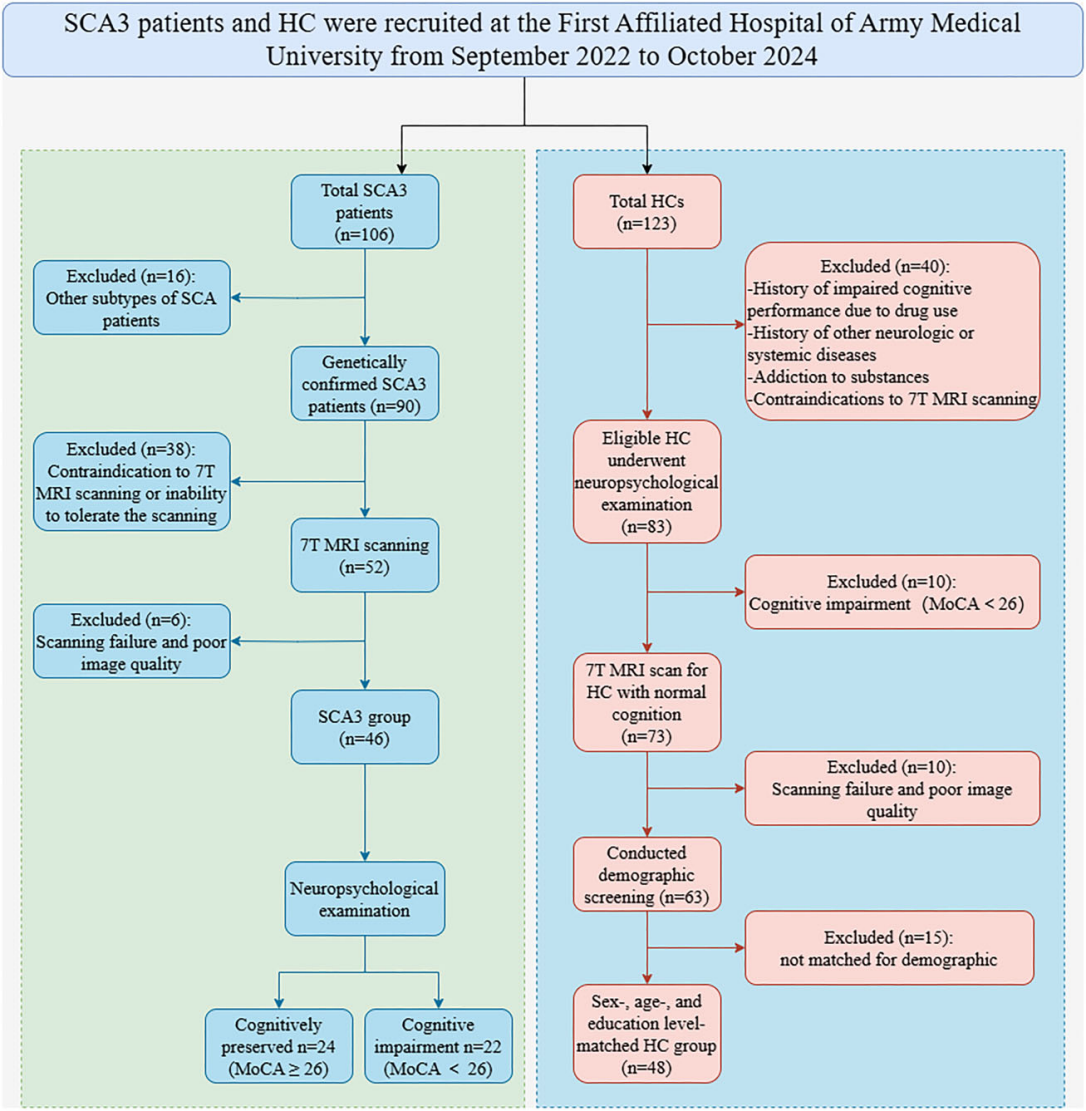
Inclusion and exclusion criteria.

Additionally, we recruited 123 volunteer healthy controls (HCs). To ensure the accuracy and reliability of the study, we excluded the following HCs during recruitment: (i) those currently taking medications that may affect cognitive performance, such as antidepressants and antipsychotics; (ii) those with histories of cranial or neurological disorders; (iii) those who scored <26 on the MoCA after adjusting for education level, indicating possible mild CI; (iv) those with histories of or current addiction to any substance, including alcohol; and (v) those who could not tolerate 7T MRI examinations or had poor image quality. Ultimately, 48 HCs matched for sex, age, and education level were included. All HCs underwent neuropsychological assessments. Both the patients and HCs were recruited from the Radiology Department of the First Affiliated Hospital of Army Medical University between September 2022 and October 2024. Figure 1 illustrates the inclusion and exclusion process, highlighting the rigorous screening applied to both SCA3 patients and HCs, ensuring cohort homogeneity for valid group comparisons.

The Medical Ethics Committee of the First Affiliated Hospital of Army Medical University approved the study (Nos: KY2020191, KY2023046). This was a clinical registry study (clinical registration numbers ChiCTR1800019901 and ChiCTR2000039434).

### MRI acquisition

All participants underwent 7T-Terra MRI (Siemens) MP2RAGE sequence scanning with a 32-channel head orthogonal coil. The scan parameters were repetition time: 4300 ms, echo time: 2.27 ms, inversion time: 1000/3200 ms, field of view: 208 × 208 mm, matrix: 320 × 300, slice thickness: 0.7 mm, slices per slab: 240, voxel size: 0.7 × 0.7 × 0.7 mm, flip angle 4.0°/4.0°, and acquisition time: 8 min 50 s. The parameters were selected to optimize the signal-to-noise ratio and anatomical contrast of small subcortical structures, while balancing scan time and minimizing motion artifacts (Shen *et al*., 2021).

### MRI data analysis

#### Preprocessing

**Figure 2:**
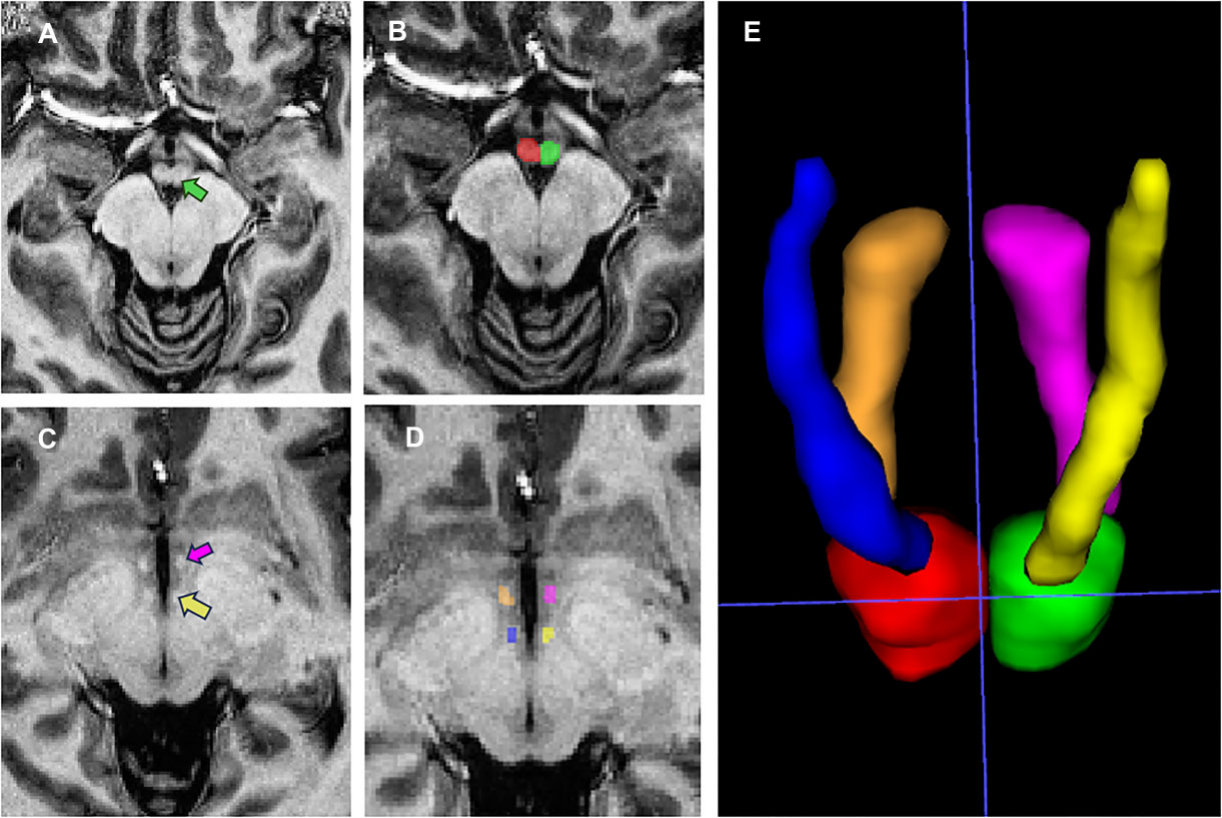
MB, MTT, and PF structures on MRI. (**A–D**) Green denotes left MB; pink denotes left PF; yellow denotes left MTT. Red, orange, and blue regions denote the right MB, PF, and MTT, respectively. (**E**) 3D view of the MB, MTT, and PF.

A radiologist with >5 years of expertise in neuroimaging used ITK-SNAP (Version 4.2.0; www.itksnap.org) software (Yushkevich *et al*., 2006) to manually segment the MB, MTT, and PF components (Fig. 2 provides a visual representation of the segmented MB, MTT, and PF on 7T MRI). Subsequently, the original images were processed into 64 × 64 × 64 voxel patches centered on these components. The input data were normalized via the Z-score method. During the training phase, various data augmentation strategies were implemented, including random flipping, translation, and scaling.

#### Segmentation model

**Figure 3:**
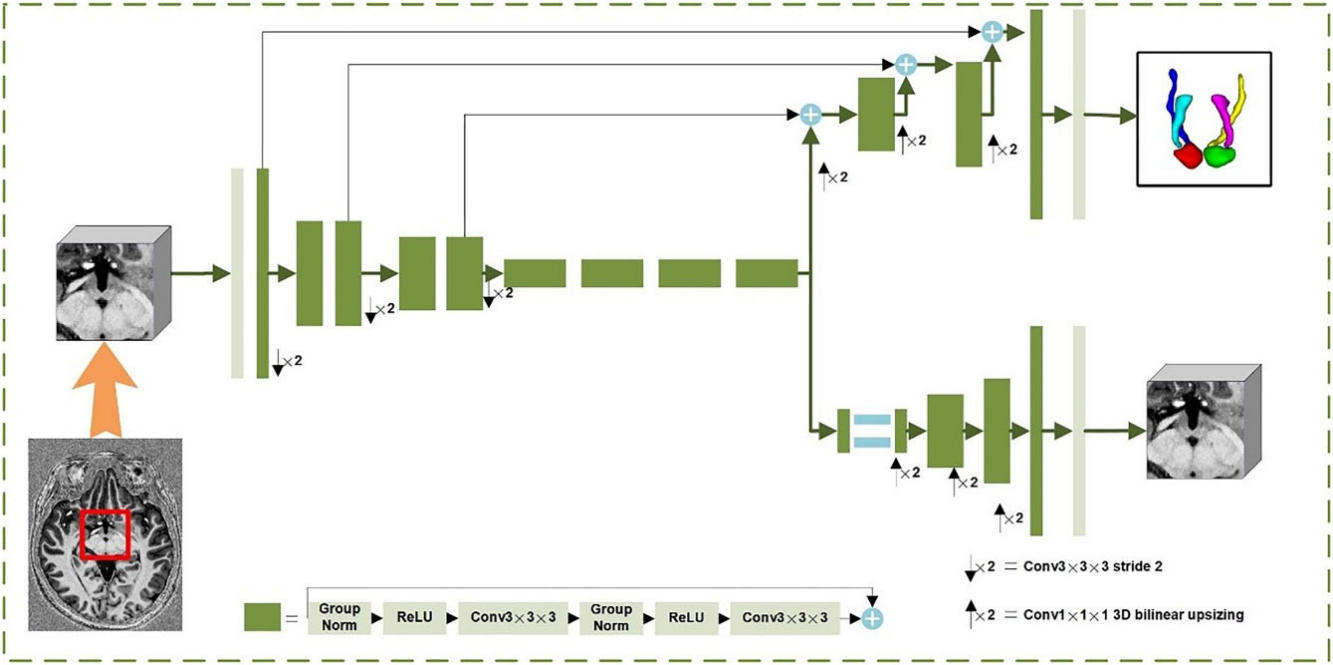
Preprocessing procedure. The structure of SegResVAE Net. The input is a cropped 3D MRI, followed by an initial 3 × 3 × 3 3D convolution with 32 filters. Each green block represents a ResNet-like block with GroupNorm normalization. The segmentation decoder outputs the predicted core components of the Papez circuit, while the VAE branch reconstructs the input image and is used solely during training to regularize the shared encoder.

SegResVAE Net6, which contains an encoder, decoder, and variational auto-encoder (VAE; Fig. 3 details the preprocessing pipeline and architecture of SegResVAE Net), was used to segment the core components mediating the Papez circuit, which processes cropped 3D MRIs through ResNet-like blocks (GroupNorm) and dual decoders: a segmentation branch for the Papez circuit and a VAE branch regularizing the encoder via input reconstruction (training only) (Myronenko, 2018). The encoder extracted the higher-order semantic information from the input images; the decoder with inter-level skip connections predicted the segment results; and the VAE reconstructed the input image dimensions, without inter-level skip connections, to add more constraints (Fig. 3).

#### Image correction and data extraction

A radiologist with >10 years of experience in neuroimaging used ITK-SNAP software to correct the results obtained from the previous two steps, and the bilateral MB, MTT, and PF volumes were then extracted and displayed in three dimensions. This hybrid approach balanced accuracy (via expert annotation) with efficiency (via automation), aligning with established practices in medical image analysis (Corballis, 2014; Willems *et al*., 2014).

### Statistical analysis

We used independent-sample t-tests to analyze the volumetric differences in the bilateral MB, MTT, and PF structures between the HC and SCA3 groups. Prior to statistical analysis, normality of key variables was assessed using Shapiro–Wilk tests. For normally distributed data (*P* > 0.05), independent-sample t-tests were used for group comparisons; non-normally distributed data (*P* ≤ 0.05) were analyzed with Mann–Whitney U tests. In correlation analyses, Pearson or Spearman coefficients were selected based on data normality. Within the SCA3 group, we conducted correlation analyses (two-tailed *P* < 0.05) to determine the relationships between the MB, MTT, and PF volumes and cognitive and memory levels (MoCA, DST, RVR, CCAS), motor abilities (ICARS, SARA), and self-care abilities (ADL, IADL). To further investigate the impact of MB structure on cognitive levels, we divided the SCA3 patients into the CI group (MoCA scores <26) and the preserved cognition (CP) group (MoCA scores ≥26) (Nasreddine *et al*., 2005). We then compared the MB, MTT, and PF volumes between these subgroups using the same statistical methods. The significance level for all statistical analyses was set at *P* < 0.05. All statistical analyses were performed using SPSS (Version 30; IBM, Chicago, IL, USA).

## Results

### Clinical characteristic comparisons

Table 1 compares the demographic and clinical characteristics of the SCA3 and HC groups. The two groups did not differ significantly in terms of sex, age, or education level (*P* > 0.05). The MoCA and DST scores were significantly lower for the SCA3 patients than for the HCs.

**Table 1:** Demographic and clinical characteristics of the SCA3 and HC groups.

	SCA3	HCs	χ²/t	*P*
Sex (male/female)	20/26	21/27	0.01	0.979
Age (years)	44 ± 11	42 ± 14	−0.948	0.346
Education (years)	12 ± 4	13 ± 4	1.773	0.079
MoCA	24 ± 5	27 ± 2	3.906	<0.01**
DST	8 ± 3	12 ± 3	5.723	<0.01**
SARA	13 ± 7	–	–	–
ICARS	37 ± 16	–	–	–
RVR	37 ± 14	–	–	–
ADL	12 ± 5	–	–	–
IADL	22 ± 10	–	–	–
HAMD	14 ± 16	–	–	–
CCAS	71 ± 21	–	–	–

SCA3: spinocerebellar ataxia type 3; HCs: healthy controls; MoCA: Montreal Cognitive Assessment, Beijing Version; DST: digit span test; RVR: rapid verbal retrieve; ADL: Activity of Daily Living Scale; IADL: Instrumental Activities of Daily Living Scale; HAMD: Hamilton Depression Rating Scale; CCAS: Cerebellar Cognitive Affective Syndrome; SARA: Scale for the Assessment and Rating of Ataxia; ICARS: International Cooperative Ataxia Rating Scale. **P < 0.01.

### Comparison of MB, MTT, and PF volumes between the SCA3 and HC groups

Based on the measurements obtained using ITK-SNAP, the MB volumes in the SCA3 group were 83.94 ± 10.49 mm^3^ on the left side and 75.47 ± 9.54 mm^3^ on the right side, compared with 96.38 ± 10.81 and 90.92 ± 10.31 mm^3^, respectively, in the HCs. The MTT volumes were 59.93 ± 8.46 mm^3^ on the left and 57.10 ± 8.10 mm^3^ on the right in the SCA3 group versus 69.92 ± 7.03 and 72.64 ± 10.52 mm^3^, respectively, in the HCs. The PF volumes were 57.81 ± 7.57 mm^3^ on both the left and right sides in the SCA3 group compared with 63.82 ± 6.62 mm^3^ on the left and 59.78 ± 7.46 mm^3^ on the right in the HCs. The SCA3 group exhibited significantly lower volumes in both the left and right MB, MTT, and PF compared with the HC group (*P* < 0.01). Table 2 quantifies the volumetric reductions in MB, MTT, and PF across SCA3 patients compared to HCs.

**Table 2:** Comparison of MB, MTT, and PF volumes between SCA3 patients and HCs.

		Mean ± SD (mm^3^)		t	*P*
		SCA3	HCs		
MB	Right	75.47 ± 9.54	90.92 ± 10.31	−2.261	<0.01**
	Left	83.94 ± 10.49	96.38 ± 10.81	−2.409	<0.01**
MTT	Right	57.10 ± 8.10	72.64 ± 10.52	−1.823	<0.01**
	Left	59.93 ± 8.46	69.92 ± 7.03	−2.621	<0.01**
PF	Right	52.78 ± 6.96	59.78 ± 7.46	−1.028	<0.01**
	Left	57.81 ± 7.57	63.82 ± 6.62	−2.210	<0.01**

MB: mammillary body; MTT: mammillothalamic tract; PF: post-commissural fornix. **P < 0.01.

### Correlations between MB, MTT, and PF volumes and cognitive and motor impairment

**Figure 4:**
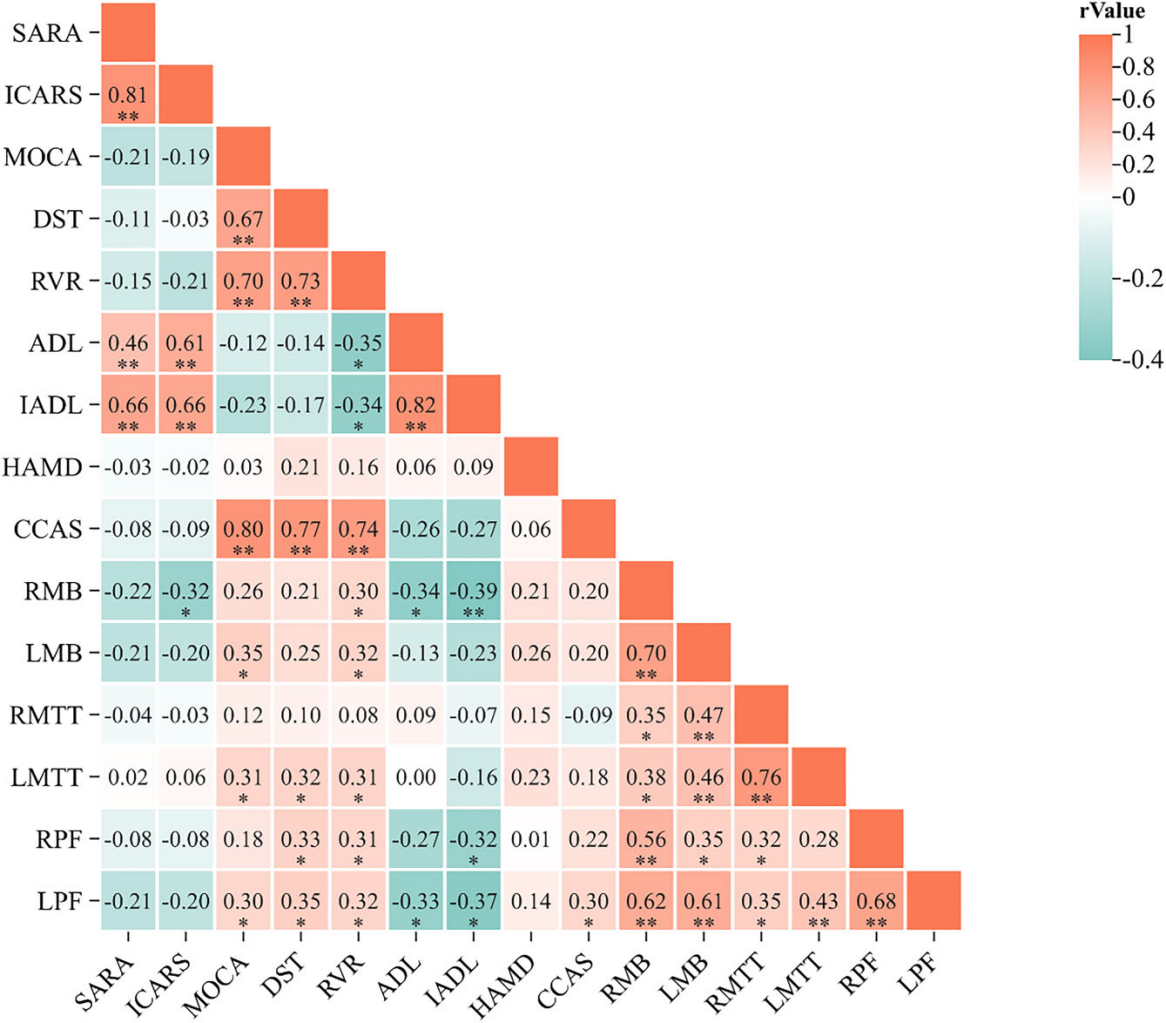
Correlation coefficient heatmap of MB, MTT, and PF volume and cognitive and motor dysfunction scores. MoCA: Montreal Cognitive Assessment, Beijing Version; DST: digit span test; RVR: rapid verbal retrieve; ADL: Activity of Daily Living Scale; IADL: Instrumental Activities of Daily Living Scale; HAMD: Hamilton Depression Rating Scale; CCAS: Cerebellar Cognitive Affective Syndrome; SARA: Scale for the Assessment and Rating of Ataxia; ICARS: International Cooperative Ataxia Rating Scale. R/LMB: right/left mammillary body; R/LMTT: right/left mammillothalamic tract; R/LPF: right/left post-commissural fornix. *P < 0.05; **P < 0.01.

Partial correlation analysis (age- and sex-corrected) indicated that in SCA3 patients, MoCA scores were positively correlated with the left MB (*r* = 0.35, *P* < 0.05), left MTT (*r* = 0.31, *P* < 0.05), and left PF (*r* = 0.30, *P* < 0.05). DST scores were positively correlated with the left MTT (*r* = 0.32, *P* < 0.05) and bilateral PF (left PF *r* = 0.33, right PF *r* = 0.35, *P* < 0.05). RVR scores were positively correlated with the left MB (*r* = 0.32, *P* < 0.05), left MTT (*r* = 0.31, *P* < 0.05), and bilateral PF (left PF *r* = 0.31, right PF *r* = 0.32, *P* < 0.05). ADL scores were negatively correlated with the right MB (*r* = −0.34, *P* < 0.05) and left PF (*r* = −0.33, *P* < 0.05). IADL scores were negatively correlated with the right MB (*r* = −0.39, *P* < 0.01) and bilateral PF (left PF *r* = −0.32, right PF *r* = −0.37, *P* < 0.05). CCAS was positively correlated with the left PF (*r* = 0.30, *P* < 0.05). ICARS was negatively correlated with the right MB (*r* = −0.32, *P* < 0.05). No remaining structures were significantly correlated with scale assessments (Fig. 4 displays the correlation heatmap between cognitive/motor scores and MB, MTT, and PF volumes).

### Neuropsychological performance and MB, MTT, and PF volumes in SCA3 patients

Within the SCA3 patients, we compared the neuropsychological tests and MB, MTT, and PF volumes between the CI and CP groups. Table 3 contrasts neuropsychological performance and structural volumes between CI and CP, highlighting left-sided structural vulnerability in CI patients. The DST, RVR, and CCAS scores differed significantly between these groups (*P* < 0.01). The SARA, ICARS, and IADL scores also differed between these groups (*P* < 0.05). The CI and CP groups did not differ significantly on the HAMD or other remaining scales (*P* > 0.05, Table 3). The bilateral MB, left MTT, and left PF volumes were significantly lower in the CI group than in the CP group (*P* < 0.05, Table 3).

**Table 3:** Neuropsychological comparison and MB, MTT, and PF volumes between CI and CP groups.

	CI (*n* = 22)	CP (*n* = 24)	t	*P*
DST	6 ± 3	10 ± 2	−4.558	<0.01**
RVR	30 ± 16	40 ± 8	−3.738	<0.01**
CCAS	57 ± 21	84 ± 10	−5.559	<0.01**
SARA	15 ± 7	11 ± 5	2.158	0.036*
ICARS	42 ± 16	32 ± 14	2.066	0.045*
ADL	13 ± 5	11 ± 4	1.559	0.126
IADL	25 ± 10	19 ± 8	2.138	0.038*
HAMD	14 ± 14	13 ± 19	0.152	0.88
RMB (mm^3^)	72.29 ± 8.57	78.38 ± 9.61	−2.261	0.029*
LMB (mm^3^)	80.24 ± 10.20	87.33 ± 9.75	−2.409	0.02*
RMTT (mm^3^)	54.89 ± 7.82	59.14 ± 7.96	−1.823	0.075
LMTT (mm^3^)	56.72 ± 8.35	62.88 ± 7.58	−2.621	0.012*
RPF (mm^3^)	51.68 ± 6.18	53.79 ± 7.6	−1.028	0.309
LPF (mm^3^)	55.34 ± 6.67	60.07 ± 7.77	−2.21	0.032*

DST: digit span test; RVR: rapid verbal retrieve; ADL: Activity of Daily Living Scale; IADL: Instrumental Activities of Daily Living Scale; HAMD: Hamilton Depression Rating Scale; CCAS: Cerebellar Cognitive Affective Syndrome; SARA: Scale for the Assessment and Rating of Ataxia; ICARS: International Cooperative Ataxia Rating Scale. R/LMB: right/left mammillary body; R/LMTT: right/left mammillothalamic tract; R/LPF: right/left post-commissural fornix.

*P < 0.05; **P < 0.01

## Discussion

To our knowledge, this study is the first to examine MB, MTT, and PF volumes in SCA3 patients using 7T MRI. We primarily investigated the specific manifestations of the MBs, MTT, and PF within the Papez circuit relative to cognitive and motor impairments in patients with SCA3. We used the MP2RAGE sequence of 7T MRI to examine structural information on the MBs, MTT, and PF in SCA3 patients and HCs and found that the MB, MTT, and PF structures differed significantly between them.

### Role of MB in cognitive function in SCA3 patients

The MBs are an important component of the hypothalamus, located on the ventral side and comprising two main nuclei: the lateral and medial nuclei (Vann & Aggleton, 2004). The MBs are commonly referred to as part of the “extended hippocampal system” (Aggleton & Brown, 1999). Studies of traumatic brain injury have shown that a history of long-term, repetitive mild traumatic brain injuries can lead to atrophy of the MBs, resulting in further memory impairment (Miyata *et al*., 2024). Research on neurodegenerative diseases such as AD has revealed that some patients with AD and mild cognitive impairment (MCI) experience reduced MB volumes (Aggleton & Brown, 1999). Additionally, structural impairments in the MBs have been observed in patients with heart failure, sleep apnea and schizophrenia (Kumar *et al*., 2008, 2009). In our study, the bilateral MBs in SCA3 patients exhibited significant atrophy compared with those of the HCs. Additionally, both MoCA and RVR scores were correlated with MB volume. MB volume was lower in SCA3 patients with CI than in those with CP, and the patients’ cognitive and memory functions were significantly associated with MB volume. Our results confirmed that SCA3 progression impacts patients’ cognitive functions and induces structural changes in the MB.

### Role of MTT and PF in cognitive function in SCA3 patients

The MTT and fornix are crucial structures connecting the MB to other brain regions and play significant roles in cognitive and memory functions. The MTT is the only white matter fiber bundle that specifically connects the MB and thalamus within the Papez circuit (Bubb *et al*., 2017). Current research indicates that damage to the MTT can lead to severe cases of amnesia, with effects that are even more pronounced than those caused by damage to the thalamic nuclei alone (Pergola, 2016; Van Der Werf *et al*., 2000). Input from the MTT to the anterior thalamic nuclei is essential for normal episodic and spatial memory. The fornix is the primary output fiber of the hippocampus and plays a significant role in connecting the medial temporal lobe to related areas (Bubb *et al*., 2017; Thomas *et al*., 2011). A previous study revealed that patients with AD and MCI often exhibit atrophy in the limbic system, and the integrity of the white matter, including the fornix, is considered partly responsible for the reduced resting-state functional connectivity in these patients (P. Wang *et al*., 2020). This is consistent with our findings. In our study, the MBs in SCA3 patients showed significant atrophy compared with those of the HCs. Concurrently, both MoCA and RVR scores were correlated with MB volume. MB volume was also lower in SCA3 patients with CI than in those with normal cognition, and patients’ motor, cognitive, and memory functions were significantly associated with MB volume. Our results confirmed that SCA3 progression impacts patients’ cognitive functions and induces structural changes in the MBs. Additionally, MB atrophy has been reported in patients with schizophrenia, heart failure, and sleep apnea. In our study, the degree of left-sided structural atrophy was more severe in the CI subgroup than in the CP subgroup. Patients’ ability to live independently may be correlated with left PF volume.

### Motor–cognitive link in SCA3 and the role of MB, MTT, and PF volume

Prior research has shown a close relationship between physical activity and cognitive ability. Exercise can augment brain volume in older adults and enhance cognitive functions encompassing executive functions and memory. Decreased motor capabilities can precipitate significant deterioration in cognitive functions. Furthermore, exercise can influence the hippocampus, thereby promoting long-term memory formation (Colcombe *et al*., 2006; Colcombe & Kramer, 2003; Keiser *et al*., 2024; Z. Wang *et al*., 2023). Our findings showed that in SCA3 patients, the right MB volume was correlated with ICARS scores, suggesting that the MB may also be involved in motor functions, a role not previously reported in MB functional studies. This finding aligns with studies showing exercise modulates Papez circuit structures: aerobic training enhances anterior thalamus and cingulate cortex gray matter (Tian *et al*., 2014), potentially through neurotrophic effects and improved cerebral perfusion (Maass *et al*., 2015). We speculate that this may indicate that, like the hippocampus, the MB has potential factors such as genes, blood flow, and metabolism that can be activated through exercise and thereby further influence patients’ cognitive functions. However, this hypothesis requires further research to validate. MB atrophy may drive SCA3 motor dysfunction via disrupted thalamocortical motor circuits. Future studies using SCA3 transgenic mice could test if MB volume loss correlates with locomotor deficits and if exercise rescues both MB integrity and motor function.

MB/MTT/PF atrophy in SCA3 presents three therapeutic opportunities: (i) volumetric loss as early biomarkers for cognitive monitoring, (ii) left-lateralized vulnerability indicating interhemispheric connectivity therapies (e.g. transcranial magnetic stimulation; X. Wang *et al*., 2024), and (iii) exercise interventions targeting hippocampal–thalamic pathways to preserve MB integrity, as evidenced in preclinical models (Keiser *et al*., 2024). These approaches require validation through longitudinal clinical trials with multimodal imaging.

The more pronounced left-sided atrophy in the MB, MTT, and PF in SCA3 patients with CI can be attributed to the left hemisphere's dominance in language and memory functions (Buckner, 2013; Habas *et al*., 2009). In neurodegenerative diseases like Alzheimer's, left-sided atrophy is often more severe, particularly in areas involved in verbal memory and executive function (Forno *et al*., 2021; Thompson *et al*., 2003). Similarly, in SCA3, the left-sided damage to the Papez circuit structures may contribute to the greater cognitive deficits observed in these patients, especially in tasks involving language and memory. This asymmetry supports the idea that left-lateralized brain regions are more vulnerable in CI.

This study has several limitations. First, we relied primarily on the MoCA and DST for cognitive assessment, which limits the comprehensiveness of our evaluation, particularly in characterizing cognitive impairment related to episodic memory. Future studies should consider incorporating a broader range of neuropsychological tests, such as the Rey Auditory Verbal Learning Test (RAVLT) or the California Verbal Learning Test (CVLT), to provide a more detailed understanding of cognitive deficits in SCA3 patients. Additionally, because severely symptomatic SCA patients could not undergo 7T MRI scans, the included patients exhibited only MCI and ataxic symptoms. This limitation constrains our ability to assess the extent of memory impairment and interpret the results. Future studies should explore alternative imaging protocols (e.g. motion-corrected sequences) or multimodal approaches (e.g. portable neuroimaging) to include broader disease stages.

## Conclusions

Our results revealed volume changes in the MB, MTT, and PF in patients with SCA3, and identified asymmetry in the atrophy of these structures. We showed that the MB pathway structures in SCA3 patients are atrophied compared with those of healthy individuals, with differences between the CI and CP subgroups. The left side exhibited earlier and more pronounced atrophy. Additionally, we discovered that motor function in SCA3 patients may affect MB structure, further influencing cognitive and memory functions. This relationship requires further investigation to validate.
